# Efficacy of a low‐carbohydrate diet combined with exercise on glycemic control and metabolic health in type 2 diabetes mellitus: A systematic review and meta‐analysis

**DOI:** 10.1111/dom.70379

**Published:** 2025-12-19

**Authors:** Ye He, Zihan Dai, Angus Pak‐hung Yu, Stephen Heung‐sang Wong, Eric Tsz‐chun Poon

**Affiliations:** ^1^ Department of Sports Science and Physical Education The Chinese University of Hong Kong Hong Kong China

**Keywords:** glucose metabolism, lifestyle intervention, meta‐analysis, T2DM

## Abstract

**Aims:**

This study aims to evaluate the efficacy of a low‐carbohydrate diet with exercise (LCD + EX) compared to a non‐carbohydrate‐restricted diet with exercise (NRD + EX) on glycemic control and other clinically relevant metabolic health outcomes in adults with type 2 diabetes mellitus (T2DM).

**Materials and methods:**

A systematic search was conducted across five databases from inception to August 1, 2025. Randomized controlled trials (RCTs) were analysed using random‐effects models, with outcomes expressed as mean difference (MD) and 95% confidence intervals (CIs).

**Results:**

Twelve RCTs involving 805 participants were included. No significant differences were observed between LCD + EX and NRD + EX in the overall analysis for glycosylated haemoglobin (MD [95% CI]: −0.18 [−0.43, 0.07] %, *p* = 0.16), fasting glucose (−0.30 [−0.67, 0.07] mmol/L, *p* = 0.11), insulin levels (−1.45 [−3.62, 0.73] μIU/mL, *p* = 0.19), and HOMA‐IR (−0.17 [−0.46, 0.11] arbitrary unit, *p* = 0.23). Additionally, no between‐group differences were observed for body composition, blood pressure, total cholesterol, and low‐density lipoprotein cholesterol levels. However, changes in high‐density lipoprotein cholesterol and triglyceride levels favoured the LCD + EX group. Subgroup analysis for intervention duration ≤6 months revealed a trend of improvement for HbA1c (−0.30 [−0.57, −0.03] %, *p* = 0.03) and fasting glucose (−0.34 [−0.69, 0.00] mmol/L, *p* = 0.05) in the LCD + EX group.

**Conclusions:**

LCD + EX did not demonstrate significant overall improvements in glycemic control or body composition compared to NRD + EX in adults with T2DM. However, potential benefits were noted in lipid profiles and in shorter interventions. Future studies can focus on differences in metabolic outcomes among various types of LCD, enabling tailored clinical recommendations.

## 1 INTRODUCTION

Type 2 diabetes mellitus (T2DM) represents a major public health challenge. There were an estimated 588.7 million adults living with diabetes worldwide in 2024, affecting 11.1% of the global population, primarily reflecting type 2 diabetes.^1^, ^2^ The core characteristic of T2DM is impaired glucose regulation, accompanied by metabolic dysregulation such as dyslipidemia.^3^ These metabolic abnormalities not only compromise metabolic health but also significantly increase the risk of cardiovascular complications.^4^ Interventions that effectively improve metabolic indicators in T2DM are crucial for slowing disease progression and reducing long‐term risks.^5^

Lifestyle interventions such as low‐carbohydrate diets and exercise represent one of the most important and actionable strategies for improving glycemic control and metabolic health in T2DM.^6^ According to the existing dietary guidelines,^7^ carbohydrates typically provide 45%–65% of total energy in a normal dietary pattern, a range considered to represent a carbohydrate‐unrestricted intake level. Consequently, diets supplying less than 45% of energy from carbohydrates can be classified as carbohydrate‐restricted, with the degree of restriction varying by intake level.^8^ Based on commonly used operational definitions from previous studies, carbohydrate restriction can be further categorized as follows: mild LCD has 26%–45% of total calories from carbohydrates and very LCD has less than 26% of total calories from carbohydrates and/or less than 130 g of carbohydrates.^8^, ^9^, ^10^ Higher intake of carbohydrate was associated with an increased risk of disease.^11^ Meanwhile, LCD has been recognized due to its capacity to directly target postprandial glycaemia, improve insulin resistance, and promote weight reduction factors that are highly relevant in the pathophysiology of T2DM.^9^, ^12^

The lack of exercise was classified as an actual cause of chronic diseases (such as T2DM and cardiovascular disease) and death.^13^ Structured exercise programs, including aerobic, resistance, high‐intensity interval training, and combined training, have demonstrated favourable outcomes on glycemic control and cardiovascular risk factors.^14^, ^15^, ^16^, ^17^ The benefits of exercise for glycemic control are largely explained by an increase in whole‐body insulin sensitivity that has been supported by previous studies indicating significant benefits in receptor affinity and muscle strength, resulting in increasing insulin sensitivity among T2DM.^18^, ^19^

By reducing insulin resistance and enhancing lipid oxidation, LCD may synergize with exercise to further optimize glycemic and body fat control.^20^, ^21^ Nevertheless, existing research predominantly focuses on isolated comparisons of LCD in contrast to other dietary patterns, including low‐fat diets and high‐carbohydrate diets,^22^, ^23^ with a scarcity of systematic investigation into the combined effects of LCD and exercise. Consequently, the comparison between exercise coupled with LCD and exercise without carbohydrate restriction is imperative to quantify the added value of LCD in enhancing exercise‐induced metabolic adaptations and establish evidence‐based protocols for optimizing T2DM interventions.

This review and meta‐analysis aims to systematically synthesize the existing evidence on the efficacy of a low‐carbohydrate diet combined with exercise (LCD + EX) compared with a non‐carbohydrate‐restricted diet with exercise (NRD + EX). Using a meta‐analytic approach, we seek to quantify the magnitude of these effects and contribute to evidence‐based clinical practice, informing future lifestyle intervention guidelines tailored for T2DM populations.

## 2 METHODS

### 2.1 Search strategy

This systematic review and meta‐analysis was conducted in accordance with the guidelines outlined in the Cochrane Handbook for Systematic Reviews^24^ and following the updated Preferred Reporting Items for the Systematic Reviews and Meta‐Analyses statement.^25^ The protocol of this review was registered at the International Prospective Register of Systematic Reviews (CRD420251032516). A systematic search was performed in the following five databases: Cochrane Central Register of Controlled Trials, EMBASE, PubMed, SPORTDiscus, and Web of Science from inception to August 1, 2025. The literature search strategy for this study utilized a combination of the following keywords and MeSH terms: (“diet, carbohydrate‐restricted” OR “low‐carbohydrate” OR ketogenic OR “dietary carbohydrate” OR “low glycemic”) AND (exercise OR training OR “physical activity” OR movement OR “exercise therapy” OR “muscle strength” OR “weight lifting” OR “circuit training” OR “resistance training” OR “isometric exercise” OR “aerobic exercise” OR “endurance training” OR “high intensity interval training” OR “moderate intensity interval training” OR “anaerobic training”). The scope of these searches was confined to human studies and full text. Randomized controlled trials (RCTs) written in English that examined the efficacy of LCD + EX for T2DM were included. In addition, secondary screening was performed by manually searching the reference lists of the resulting literature for additional access to relevant studies. The specific search strategy is reported in Table S1, Supporting Information.

### 2.2 Selection procedure and eligibility criteria

The inclusion criteria were developed using the population, intervention, comparison, outcomes, and study type (PICOS) framework. Specifically, studies were included if they involved adults diagnosed with T2DM, defined according to established criteria.^26^, ^27^ Participants could be of any sex, aged 18 years or older, and may or may not have comorbid conditions, provided that T2DM was the primary diagnosis of interest. Studies focusing exclusively on type 1 diabetes, gestational diabetes, or prediabetic individuals were excluded. In terms of intervention, RCTs of a combined low‐carbohydrate diet plus any exercise were eligible for inclusion. LCD was defined as a dietary pattern in which carbohydrate intake constituted less than 45% of total daily energy intake, while allowing ad libitum intake of calories, fat, and protein.^28^, ^29^, ^30^ Participants in the comparator group (NRD + EX) maintained their usual dietary habits and performed the same exercise as the intervention group throughout the study period. NRD was defined as 45%–60% of total calories from carbohydrates without imposing limits on fat or protein.^8^, ^28^ All types of exercise would be included in the studies. Glycemic control represents the direct therapeutic targets of the interventions, which is the most clinically relevant indicator for diabetes prevention and management. In addition, body composition, blood pressure, and lipid profiles can provide complementary information on metabolic health. Accordingly, the primary outcomes in this study were glycemic parameters, including glycosylated haemoglobin (HbA1c), fasting glucose, insulin levels, and homeostatic model assessment for insulin resistance (HOMA‐IR). Secondary outcome measures related to cardiometabolic health, including body composition parameters such as body weight, body mass index (BMI), waist circumference, body fat, fat‐free mass, as well as blood pressure, total cholesterol (TC), low‐density lipoprotein cholesterol (LDL‐C), high‐density lipoprotein cholesterol (HDL‐C), and triglycerides (TG), were also evaluated in the analyses. This systematic review and meta‐analysis only included RCTs to ensure a high level of evidence and minimize potential bias. Studies with other designs, such as observational studies, case series, or non‐randomized trials, were excluded.

### 2.3 Data extraction

The data was extracted by two independent reviewers (Y.H. and E.P.) using a standardized, well‐structured Microsoft Excel spreadsheet according to the pre‐established guidelines. The extracted content includes: basic information of the included studies, such as author, publication year, and research country/region; characteristics of the subjects, including sample size, gender ratio, average age, and BMI; characteristics of the intervention, including the criteria for carbohydrate intake, proportion of fat and protein, as well as the range of total energy intake; intervention duration and exercise protocol. Conflicts were resolved through discussion with a third reviewer (S.W.). If the original data is not provided, attempts will be made to contact the author for supplementation, and exclusions will be undertaken based on the guidelines of systematic reviews if the connection is unsuccessful. All data were organized in Microsoft Excel using uniform units for subsequent quantitative synthetic analyses.

### 2.4 Risk of bias assessment and overall certainty of evidence

Two independent authors (Y.H. and E.P.) used the revised Cochrane Risk of Bias Tool (RoB 2)^31^ to evaluate the risk of bias in the included studies. This tool assesses the quality of studies in five domains: randomization process, deviations from the intended interventions, missing outcome data, bias in outcome measurement, and bias in outcome reporting. Each of these domains was assessed as “low risk,” “some concerns,” or “high risk.” In addition, the assessment summarized each domain's result into one overall level. The overall quality of evidence was assessed by the Grading of Recommendations Assessment, Development, and Evaluation (GRADE).^32^ Based on five criteria: risk of bias, inconsistency, indirectness, imprecision, and publication/reporting bias, the certainty of evidence was classified into four levels: high, moderate, low, or very low. Any disagreements or discrepancies during the assessment were resolved through discussion with a third reviewer (S.W.).

### 2.5 Data synthesis and analysis

This study was based on Review Manager software (RevMan V.5.4; Cochrane Collaboration, Oxford, UK) for full data integration and statistical analysis. Articles that reported at least one relevant outcome of interest were included. To ensure the interpretability of pooled results, meta‐analysis was performed when at least three studies reported the same outcome.^33^ Our analytical approach aligns with the guidance provided in the Cochrane Handbook for Systematic Reviews of Interventions.^24^ For the comparison of the effects of LCD + EX versus NRD + EX, all effect sizes were calculated directly from the mean and standard deviation of the post‐intervention measurements using random‐effects models along with MD and 95% CIs. All outcomes were converted into consistent units before analysis, ensuring comparability across studies. Alternatively, if no post‐intervention data were reported in the literature, the fraction of change from baseline to endpoints was used for the estimation (mean and standard deviation of the change must be provided explicitly in the original article). Each included study contributed only one pair‐wise comparison to the overall analysis to correct for the unit‐of‐analysis error.^24^

The heterogeneity of the studies was quantified by the *I* ^2^ statistic: *I* ^2^ ≤ 25%, 50% and 75% was considered low, moderate, and high heterogeneity, and >75% suggested a significant source of heterogeneity.^24^ A sensitivity analysis was performed in the primary outcome using one study exclusion at a time to systematically assess the extent to which a single study contributed to the heterogeneity.^34^ In order to explore the potential impact of the intervention duration, subgroup analysis was performed based on the study duration (>6 months or ≤6 months).^35^ Each subgroup consisted of at least three studies. Statistical significance was determined by a two‐tailed test (*α* = 0.05), and the effect size calculation and data transformation processes were independently verified by two authors and strictly followed the standardized operational procedures of the Cochrane Handbook.^24^

## 3 RESULTS

### 3.1 Study selection

**FIGURE 1:**
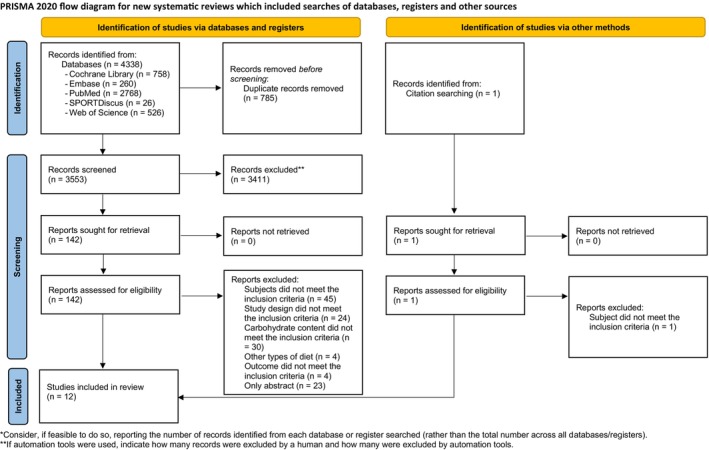
PRISMA 2020 Flowchart of included studies. *Source*: Page et al.^69^

Following a systematic database search, a total of 4338 records were initially identified. After removing duplicates, 3553 articles remained for title and abstract screening. Subsequently, 142 articles were assessed for full‐text eligibility, resulting in 12 studies meeting the predefined inclusion criteria for analysis (Figure 1).^21^, ^36^, ^37^, ^38^, ^39^, ^40^, ^41^, ^42^, ^43^, ^44^, ^45^, ^46^

### 3.2 Study characteristics

A detailed overview of the baseline characteristics of the included studies can be found in Table 1. The published year of the included studies was from 2014 to 2025. A total of 805 participants were included in this systematic review and meta‐analysis. The mean age of T2DM participants was 57 years old. Among the included studies, all provided detailed information on macronutrient consumption with calorie restriction, except for two studies,^39^, ^45^ where the carbohydrate‐restricted group reached 100% of the estimated energy requirement. One article employed high‐intensity interval exercise as the training modality, one utilized a multicomponent training approach, and nine articles utilized an aerobic or plus resistance training approach. The intervention duration in the included studies was from 8 weeks to 2 years.

**TABLE 1:** Baseline characteristics of the included studies.

Author	Population (health status, *N*, sex)	Study duration (weeks)	Group	Sample size	Age (year)	BMI	Diabetes (years)	Carbohydrate % (g)	Fat % (g)	Protein % (g)	Energy (kcal)	Exercise protocol
Asle et al.,^36^ (Iran)	Obese men with T2DM; *N* = 42	24	LCD‐V + EX	11	47.1 ± 7.0	33.0 ± 1.6	6	20	45	35	1800 kcal/day	Weekly 60‐min HIIT sessions included a 5‐min warm‐up, 10 intervals (15 s at 75% HRmax, 15 s at 85%, 30 s at 90%), active rest (30–40% HRmax), and a 5‐min cool‐down. Intensity increased every 4 weeks. Target Borg RPE was 18 (12 weeks)
			LCD‐M + EX	11	44.7 ± 7.6	33.4 ± 2.4	7	30	60	10		
			NRD + EX	11	47.1 ± 7.0	35.2 ± 3.5	7	50	30	20		
			NRD + EX	9	44.8 ± 6.2	33.2 ± 2.4	8	50	35	15		
Brinkworth et al.^37^ (Australia)	Obese adults with T2DM, *N* = 115	52	LCD‐V + EX	58	58.5 ± 7.1	34.6 ± 4.3	NR	14 (<50 g)	58	28	~6 to 7 MJ (energy‐restricted)	60‐min of combined aerobic and resistance exercise, 3 days/week
			NRD + EX	57				53	30	17		
Han et al.^21^ (China)	Men and women with T2DM, *N* = 121	24	LCD‐V + EX	60	51.5 ± 13.4	24.5 (22.7–27.3)	3.0 (0.3–8.0)	14 (<50 g)	58	28	~1,796 kcal/day (isoenergetic across diets)	Moderate‐intensity aerobic and resistance exercise, 3 days/week, 60 min/session
			NRD + EX	61				53	30	17		
Kakoschke et al.^38^ (Australia)	Men and women with T2DM, *N* = 115, (43% women)	2 year	LCD‐V + EX	58	58.5 ± 7.1	34.6 (SE = 0.4)	NR	14 (<50 g)	58	28	~500 to 1000 kcal/day deficit	Aerobic/resistance exercise (1 h, 3 days/week)
			NRD + EX	57				53	30	17		
Kindlovits et al.^39^ (Portugal)	Men and women with T2DM, *N* = 42, (25 women)	8	LCD‐M + EX‐HYPOXIA	14	72.2 (4.0)	29.0 (3.8)	NR	40	40	20		(1) A 3000 m simulated altitude; 75%HR reserve; 40‐min of moderate aerobic exercise, alternated every 9‐min on a cycle ergometer and a treadmill with 1‐min rest between them. (2) Alternately between weeks, three 15‐min strength exercises, using 3 series of 12 to 15 repetitions per exercise, with a 1‐min rest between sets
			NRD + EX‐HYPOXIA	14				60	20	20		
			NRD + EX‐NORMOXIA	14				60	20	20	100% of the estimated energy requirement	
Kindlovits et al.^45^ (Portugal)	Men and women with T2DM, *N* = 42, (25 women)	8	LCD‐M + EX‐HYPOXIA	14	70.7 (4.0)	29.3 (3.4)	NR	40	40	20	100% of the estimated energy requirement	Each session lasted ~60 min, 3 times/week, moderate‐intensity aerobic exercise between a cycle ergometer and a treadmill, with strength exercises alternated weekly
			NRD + EX‐HYPOXIA	14	71.6 (3.8)	28.3 (4.0)		60	20	20		
			NRD + EX‐NORMOXIA	14	74.4 (3.6)	29.4 (4.1)		60	20	20		
Rock et al.^40^ (United States)	Men and women with T2DM, *N* = 227, (116 women)	48	LCD‐M + EX	40M,37F	57.3 (8.6)	36.2 (4.7)		45	30	25	1200 to 2000 kcal/day	Increased physical activity was encouraged, with the goal of 30 min of physical activity on 5 days/week
			NRD + EX	39M,35F	55.5 (9.2)	36.2 (4.3)	NR	60	20	20		
			NRD	32M,44F	56.8 (9.3)	36.3 (4.4)		55	30	15		
Struik et al.^41^ (Australia)	Men and women with T2DM, *N* = 84, (40 women)	16	LCD‐V + EX	41	58.7 ± 6.6	34.5 ± 4.1	6.7 ± 5.6	14 (<50 g)	58	28		Progressive multicomponent exercise (60 min; 3 days/week)
			NRD + EX	43				53	30	17	Energy restricted (500–1000 kcal/day deficit)	
Tay et al.^42^ (Australia)	Obese adults with T2DM, *N* = 115 (43% women)	24	LCD‐V + EX	58	58 ± 7	34.4 ± 4.2	NR	14 (<50 g/days)	58	28		Supervised 60‐min structured exercise classes on 3 non‐consecutive days per week, incorporating moderate‐intensity aerobic/resistance exercises
			NRD + EX	57				53	30	17	1429 kcal/day	
Tay et al.^43^ (Australia)	Obese adults with T2DM, *N* = 115 (38 withdrawals)	52	LCD‐V + EX	41	58 ± 7	34.6 ± 4.3	8 ± 6	14 (<50 g)	58	28	1429 kcal/day	Supervised aerobic and resistance exercise (60 min; 3 days/week)
			NRD + EX	37				53	30	17		
Tay et al.^44^ (Australia)	Obese adults with T2DM, *N* = 115 (43% women)	96 (2 years)	LCD‐V + EX	58	58 (56–60)	34.2 (33.1–35.3)	6 (4–7)	14 (<50 g)	58	28	1429 kcal/day	Supervised 60‐min exercise classes, incorporating moderate‐intensity aerobic and resistance exercise on 3 non‐consecutive days per week
			NRD + EX	57	58 (56–60)	35.1 (34.0–36.2)	8 (6–10)	53	30	17		
Wycherley et al.^46^ (Australia)	Obese adults with T2DM, *N* = 115	48	LCD + EX	58	58 ± 7	34.6 ± 4.3	NR	14	58	28	1429 kcal/day	Exercise (60 min, 3/week)
			NRD + EX	57				53	30	17		

Abbreviations: EX, exercise; F, female; HIIT, high‐intensity interval training; HR, heart rate; LCD, low‐carbohydrate diet; LCD‐M, moderate low‐carbohydrate diet; LCD‐V, very low‐carbohydrate diet; M, male; NR, not reported; NRD, non‐carbohydrate‐restricted diet; T2DM, type 2 diabetes mellitus.

Adherence monitoring was reported in most of the included studies, although the methods used to assess participants' compliance with dietary and exercise interventions varied considerably. Common strategies included self‐reported dietary intake records, exercise logs, session attendance tracking, and objective measures such as direct supervision. A summary of adherence monitoring methods across the included studies is presented in Table 2. Key dietary recording methods include self‐record, software monitoring online, and prepared meals. Strategies for promoting adherence included consultations, economic benefit (food voucher), and food provision.

**TABLE 2:** A summary of adherence monitoring methods of the included studies.

Study	Diet recording method	Dietary compliance assessment	Exercise protocol	Exercise intensity monitoring	Adherence strategies in study design
Asle et al.^36^	Self‐report every 3 days; standard meal plans provided	Check regularly with a physician report to the physician, who reviewed and adjusted the plan as needed	HIIT on cycle ergometers, 3 days/week (10 intervals of 1 min each)	Heart rate monitoring (75–90% HRmax); supervised by physicians	Regular dietitian guidance; standardized recipes; rescheduled sessions if glucose levels were unstable
Brinkworth et al.^37^	Prescriptive meal plans with specific macronutrient profiles; dietitian consultations	Fortnightly dietitian reviews; partial provision of pre‐packaged foods (30% of total energy)	Combined aerobic and resistance training, 3 days/week (60 min/session)	Supervised by exercise professionals; training logs maintained	Free key foods or food vouchers; fortnightly/monthly dietitian support; supervised exercise sessions
Han et al.^21^	CDC Nutrition Calculator V2.63 software for real‐time dietary tracking	Software‐generated nutrient analysis; exclusion of non‐compliant participants	Moderate aerobic and resistance training, 3 days/week (60 min/session)	Supervised by professionals; recorded exercise duration and intensity	Monthly follow‐ups, detailed recipes and meal plans; software‐based monitoring; exclusion criteria
Kakoschke et al.^38^	Participants followed a structured dietary plan, with meal plans and recipes provided by a dietitian for the first 12 weeks	Bi‐weekly dietary monitoring for the first 12 weeks, followed by monthly assessments; food provisions were also supplied	Primarily aerobic exercise, including brisk walking and running, combined with strength training	Participants recorded their exercise activities, and researchers periodically reviewed and provided feedback	High‐frequency supervision in the initial 12 weeks, followed by food support and scheduled follow‐ups to sustain adherence
Kindlovits et al.^39^	Personalized meal plans via Dietbox® software, with recipes and nutritional guidance	Weekly 24‐h dietary recall (carbohydrate/fat intake), regular appointment	Three 1‐h sessions/week of alternating aerobic cycling, treadmill training, and core/limb strength exercises	Hypoxic chamber sessions with pulse oximetry (oxygen saturation/heart rate); Borg's RPE scale	Free transportation; fixed weekly exercise schedules; biweekly dietitian sessions (first 8 weeks)
Kindlovits et al.^45^	Personalized dietary plan created using the Dietbox® software	Weekly 24 h recalls, two individual appointments with a nutritionist during the 8‐week intervention	Each session lasted approximately 60 min, 3 times/week, moderate‐intensity aerobic exercise between a cycle ergometer and a treadmill, with strength exercises alternated weekly	Normoxia or hypoxia (simulating an altitude of 3000 m through nitrogen dilution) with heart rate and oxygen saturation monitored; Borg's RPE scale	Chauffeured transportation, regular meetings
Rock et al.^40^	Pre‐packaged meals (7 days/week for the first 6 months, then 5 days/week); participants recorded food intake and activity	Meal consumption tracking; plasma carotenoids (fruit/vegetable intake)	≥5 days/week of 30‐min moderate‐intensity activity (e.g., walking, swimming)	Self‐reported via Godin Leisure‐Time Exercise Questionnaire	Weekly behavioural counselling; free pre‐packaged meals; online education/support platform; regular phone/email follow‐ups
Struik et al.^41^	Semi‐quantitative daily food records were used to track dietary intake	Food packages were provided every 2 months. Participants received AUD 50 vouchers to enhance adherence	Moderate‐intensity aerobic exercise	Initial detailed guidance was provided, followed by regular check‐ins via phone or in person	Material incentives (food packages, vouchers) and regular communication with researchers
Tay et al.^42^	Daily weighed food records (7 days, including 2 weekend days) analysed via foodworks	Urinary urea‐to‐creatinine ratio (protein intake). Plasma β‐hydroxybutyrate (carbohydrate restriction)	Supervised 60‐min structured exercise sessions (3×/week): aerobic + resistance training	Triaxial accelerometry (ActiGraph GT3X+) to measure activity counts and MVPA time	Biweekly dietitian meetings for 12 weeks, then monthly. Provided key foods (~30% energy). Free exercise classes and food vouchers
Wycherley et al.^46^	Daily weighed food records analysed similarly, with continued monitoring over 52 weeks	Biomarkers: urinary urea‐to‐creatinine ratio and plasma β‐hydroxybutyrate	Supervised exercise program (3×/week): aerobic + resistance training	Accelerometry used similarly to monitor physical activity levels and adherence	Continued structured dietitian support and exercise supervision. Ongoing food provisions and vouchers

Abbreviations: HIIT, high‐intensity interval training; HRmax, maximum heart rate; MVPA, moderate‐to‐vigorous‐intensity physical activity; RPE, rating of perceived exertion.

### 3.3 Meta‐analysis

#### 3.3.1 Effects on glycemic control

**FIGURE 2:**
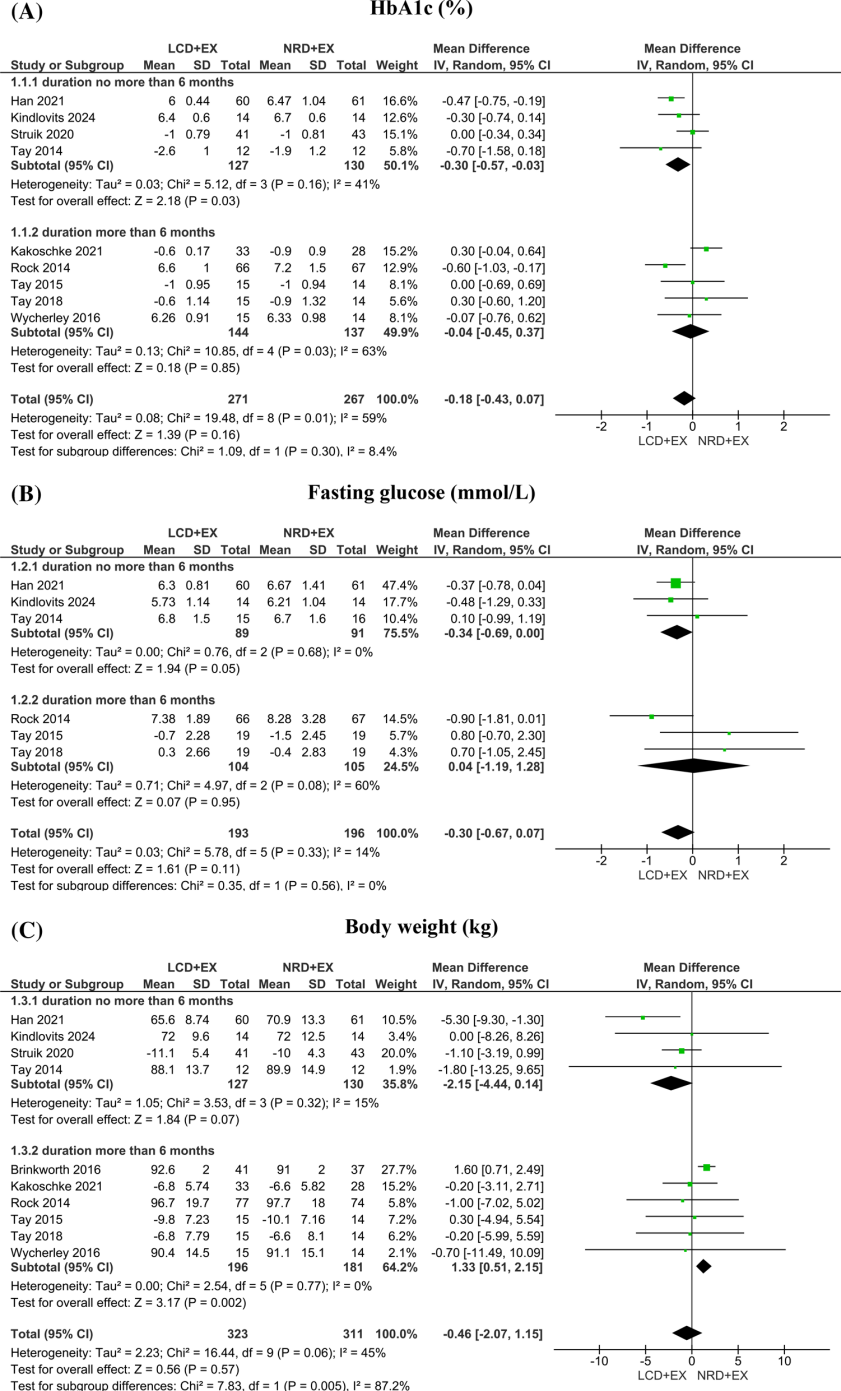
Forest plot of the effects of LCD + EX and NRD + EX on (a) HbA1c (expressed in MD with 95% CI), (b) fasting glucose (expressed in MD with 95% CI), and (c) body weight (expressed in MD with 95% CI).

A total of nine RCTs^21^, ^38^, ^39^, ^40^, ^41^, ^42^, ^43^, ^44^, ^46^ involving 538 participants reported changes in HbA1c levels. The pooled analysis showed no statistically significant difference in HbA1c between LCD + EX and NRD + EX (MD [95% CI]: −0.18 [−0.43, 0.07] %; *p* = 0.16), with high heterogeneity observed (*I* ^2^ = 59%). However, sensitivity analysis revealed that after excluding one study,^38^ not only altered statistical significance (MD [95% CI]: −0.28 [−0.48, −0.07] %; *p* = 0.009) but also reduced heterogeneity (*I* ^2^ = 29%) (Table S3). A subgroup analysis was conducted based on intervention duration. Intervention duration less than or equal to 6 months showed statistically significant improvement in HbA1c (MD [95% CI]: −0.30 [−0.57, −0.03] %; *p* = 0.03) (Figure 2a).

Fasting glucose was assessed in six articles^21^, ^39^, ^40^, ^42^, ^43^, ^44^ with a combined sample size of 389 participants. The comparison between LCD + EX and NRD + EX indicated that there was no significant difference (MD [95% CI]: −0.30 [−0.67, 0.07] mmol/L; *p* = 0.11). There was low heterogeneity among the included studies (*I* ^2^ = 14%). A sensitivity analysis after removing one study^43^ revealed statistical significance (MD [95% CI]: −0.38 [−0.69, −0.06] mmol/L; *p* = 0.02) with low heterogeneity (*I* ^2^ = 0%), indicating that the pooled estimate is sensitive to this study (Table S3). The result of subgroup analysis is close to significant in intervention duration less than or equal to 6 months in fasting glucose (MD [95% CI]: −0.34 [−0.69, 0.00] mmol/L, *p* = 0.05) with low heterogeneity (*I* ^2^ = 0%). Full results are shown in Figure 2b.

Outcome related to insulin levels was reported by four articles^40^, ^42^, ^43^, ^44^ with 240 participants included. There was no statistically significant difference between groups (MD [95% CI]: −1.45 [−3.62, 0.73] μIU/mL; *p* = 0.19) with low heterogeneity (*I* ^2^ = 0%) (Table S2). In addition, four studies^39^, ^42^, ^43^, ^44^ included 135 participants and reported no HOMA‐IR difference between groups (MD [95% CI]: −0.17 [−0.46, 0.11]; *p* = 0.23) (Table S2). The sensitivity analysis of insulin and HOMA‐IR was shown in Table S3.

#### 3.3.2 Effects on body composition

There were 10 included articles^21^, ^37^, ^38^, ^39^, ^40^, ^41^, ^42^, ^43^, ^44^, ^46^ that reported body weight in 634 individuals. There was no difference between groups, which revealed moderate heterogeneity (MD [95% CI]: −0.46 [−2.07, 1.15] kg; *p* = 0.57; *I* ^2^ = 45%). Subgroup analyses based on intervention duration were conducted with a significant difference in the duration of more than 6 months (MD [95% CI]: 1.33 [0.51, 2.15] kg; *p* = 0.002; *I* ^2^ = 0%). Detailed results are presented in Figure 2c. A total of 407 participants included in six articles^21^, ^39^, ^40^, ^42^, ^43^, ^44^ reported BMI as the outcome. There was no statistical difference in BMI between groups with moderate heterogeneity (MD [95% CI]: −0.54 [−1.51, 0.42] kg/m^2^; *p* = 0.27; *I* ^2^ = 43%). Subgroup analysis based on intervention duration did not reveal any statistically significant interactions (Table S2). There was no significant effect on waist circumference, body fat, and fat‐free mass (Table S2). Sensitivity analyses using the leave‐one‐out method did not reveal a substantial impact of individual studies on any body composition results (Table S3).

#### 3.3.3 Effects on blood pressure and lipid profiles

Systolic blood pressure and diastolic blood pressure outcomes were assessed in five studies^40^, ^42^, ^43^, ^44^, ^45^ with 270 participants. The pooled analysis indicated no significant group difference for either systolic (MD [95% CI]: −0.36 [−3.89, 3.18] mmHg; *p* = 0.84; *I* ^2^ = 0%) or diastolic blood pressure (MD [95% CI]: 0.11 [−2.08, 2.30] mmHg; *p* = 0.92; *I* ^2^ = 0%) (Table S2).

Five studies^40^, ^42^, ^43^, ^44^, ^45^ reported lipid‐related outcomes, including TC, HDL‐C, LDL‐C, and TG. The pooled analyses revealed no statistically significant differences between groups across TC (MD [95% CI]: −0.04 [−0.27, 0.20] mmol/L; *p* = 0.75; *I* ^2^ = 0%) and LDL‐C (MD [95% CI]: −0.02 [−0.24, 0.19] mmol/L; *p* = 0.84; *I* ^2^ = 0%). There were no significant effects on TC and LDL‐C of the pooled analysis. However, significant between‐group differences were detected in HDL‐C and TG. Specifically, LCD + EX was associated with higher HDL‐C compared to NRD + EX, with low heterogeneity (MD [95% CI]: 0.07 [0.01, 0.14] mmol/L; *p* = 0.03; *I* ^2^ = 0%). Additionally, TG levels were lower in LCD + EX than in NRD + EX, also showing low heterogeneity (MD [95% CI]: −0.23 [−0.43, −0.04] mmol/L; *p* = 0.02; *I* ^2^ = 0%) (Table S2).

### 3.4 Risk of bias and overall certainty of evidence

The summary assessment of risk of bias for the included studies is shown in Table 3. There were two articles at high risk of bias, and 10 articles had some concerns. The high risk of bias was related to convenience sampling, an unclear randomization method, and allocation concealment in the assessment of the randomization domain. There was a high risk in the assessment of deviations from intended interventions, which is related to dietary adherence not objectively measured; commercial program providers were unblinded, which had potential for performance bias. In addition, the high attrition and uneven dropout across groups are related to the high risk in the assessment of missing outcome data. Based on the GRADE evidence profile (Table S4), most pooled results were rated as very low‐quality evidence, with the other three outcomes classified as low. The downgrading of evidence quality was primarily due to study bias, inconsistent results, and imprecise confidence intervals, while no significant concerns regarding indirectness were identified.

**TABLE 3:** Risk of bias assessment (RoB 2).

	Randomization process	Deviations from intended interventions	Missing outcome data	Measurement of the outcome	Selection of the reported result	Overall bias
Asle et al.^36^	Some concerns	High	Some concerns	Some concerns	Low	High
Brinkworth et al.^37^	Low	Some concerns	Low	Low	Some concerns	Some concerns
Han et al.^21^	Low	Some concerns	Low	Some concerns	Low	Some concerns
Kakoschke et al.^38^	Low	Some concerns	Some concerns	Some concerns	Low	Some concerns
Kindlovits et al.^39^	Low	Some concerns	Low	Low	Low	Some concerns
Kindlovits et al.^45^	Low	Some concerns	Low	Low	Low	Some concerns
Rock et al.^40^	Low	High	Some concerns	Some concerns	Some concerns	High
Struik et al.^41^	Low	Some concerns	Low	Some concerns	Low	Some concerns
Tay et al.^42^	Low	Some concerns	Low	Low	Low	Some concerns
Tay et al.^43^	Low	Some concerns	Some concerns	Some concerns	Low	Some concerns
Tay et al.^43^	Low	Some concerns	Some concerns	Some concerns	Low	Some concerns
Wycherley et al.^46^	Low	Some concerns	Some concerns	Some concerns	Low	Some concerns

## 4 DISCUSSION

To the best of our knowledge, this systematic review and meta‐analysis is the first to summarize the effects of combining LCD + EX compared to NRD + EX in adults with T2DM. The outcomes of the study focused on glycemic control, with other clinically relevant metabolic markers, aiming to comprehensively explore the additional effects of LCD + EX.

### 4.1 Effects on glycemic control

The results of the meta‐analysis indicated that the intervention of LCD + EX did not show a significant difference in improving HbA1c, fasting glucose, fasting insulin, and HOMA‐IR compared with the intervention of exercise combined with a diet that did not limit carbohydrate intake. Variability in caloric intake and macronutrient proportions across dietary instructions in the included studies may have influenced the overall effects observed.

Sensitivity analyses were performed using the leave‐one‐out method to examine the robustness of the findings. Notably, after removing the study by Kachosche et al.,^38^ a significant additional effect on HbA1c was observed between the two groups. This may be attributed to participants' reduced adherence and behavioral adaptation over the 2‐year study period. Additionally, the sensitivity analysis indicated that excluding the study by Tay et al.^43^ could lead to significant differences in fasting glucose levels. This may be due to the calorie deficit in both groups that have masked the effects of carbohydrate restriction. Sensitivity analysis for insulin level and HOMA‐IR did not materially alter the pooled estimates, suggesting the robustness of the results.

Moreover, the subgroup analysis shows that in studies with an intervention duration of no more than 6 months, the reduction in HbA1c was more remarkable compared to studies with an intervention duration longer than 6 months. This finding is consistent with a previous review,^47^ indicating that carbohydrate restriction leads to earlier improvement in glucose control and a slight fluctuation in the later stage, accompanied by the practical limitations of the LCD, such as food choice and long‐term adherence. In addition, subgroup analysis reveals that the results in duration of no more than 6 months show a marginally significant difference between LCD + EX and NRD + EX in fasting glucose. Adopting LCD can reduce the supply of exogenous glucose and may stimulate the body's adaptive regulation in the short term, thereby improving fasting glucose.^48^

### 4.2 Effects on body composition

Compared with NRD + EX, LCD + EX shows no significant difference in improving body weight, BMI, waist circumference, body fat, and fat‐free mass. There were differences in energy deficits among subjects in different studies, which may weaken the overall effect of LCD on body weight.^49^, ^50^, ^51^ This is consistent with previous analyses, suggesting that the effect of LCD on weight management may be limited by factors such as energy intake regulation, adherence maintenance, and metabolic adaptation.^52^, ^53^ On the other hand, similar energy expenditure generated by exercise may have narrowed the difference in energy balance between the LCD + EX and NRD + EX, which might be the reason for the lack of significant differences between these two groups.^54^

Nevertheless, after removing the study of the 6‐month intervention^21^ in sensitivity analysis, the pooled results reached statistical significance, which reveals greater weight reduction in NRD + EX. In addition, subgroup analysis with intervention duration more than 6 months shows a significantly lower body weight in the NRD + EX. It is worth noting that long‐term LCD may lead to metabolic adaptive changes in the body when adapting to carbohydrate restriction, especially in the balance between fat oxidation and storage.^55^

### 4.3 Effects on lipid profiles and blood pressure

Lipid profiles have always been a key focus of studies in metabolic health. Our pooled results indicate that the LCD + EX has greater improvement in TG and HDL‐C compared to NRD + EX, while changes in TC and LDL‐C did not reach statistical significance. This finding is consistent with previous studies that LCD did not show significant differences in LDL‐C levels and TC, but had better effects on HDL‐C and TG compared to another diet without carbohydrate restriction.^22^, ^56^, ^57^

This discrepancy may be attributed to differences in the mechanisms by which LCD regulates lipid metabolism pathways. The ameliorative effects of LCD on TG and HDL‐C may primarily be attributed to its modulatory effect on lipid metabolism. A substantial diminution in the consumption of carbohydrates prompts the body to transition into a state of ketone production, at which juncture adipose tissue becomes the predominant source of energy, thereby amplifying fat mobilization and fatty acid oxidation.^58^ This process decreases triglyceride synthesis in the liver and LDL‐C secretion, ultimately leading to a significant decrease in TG levels and improving HDL‐C functionally.^59^, ^60^, ^61^ Although the participants in this study had metabolic abnormalities, no between‐group difference was observed in LDL‐C and TC levels, which may be influenced by multiple factors. For instance, the types and quantities of calories, fats, and proteins consumed were not uniformly controlled, which may have weakened the potential beneficial effects of LCD on LDL‐C.^49^, ^62^

Our pooled results show no significant difference in either systolic or diastolic blood pressure, which may be related to the complex regulatory mechanisms governing blood pressure. Beyond the differing effects of other nutrients in the diet on blood pressure,^63^ exercise enhances cardiopulmonary function and reduces arterial stiffness, thereby preventing the insidious rise in systolic blood pressure and the development of hypertension.^64^ Changes in blood pressure varied among studies, and no evidence shows LCD is more effective than unrestricted carbohydrate diets, such as a high‐carbohydrate diet.^65^

### 4.4 Methodological issues of included RCTs

The risk of bias in these studies mainly lies in the subjective factors in the data collection process, such as participants' failure to fully adhere to the dietary intervention or incomplete reporting of results, which may lead to biases in outcome measurement. Although some studies indicated that LCD + EX had modest effects in improving glycemic control in the short term, caution is still needed when interpreting these results, especially in terms of generalizability when considering the potential bias in some studies.

Furthermore, in interpreting the findings of this meta‐analysis, it is important to consider the heterogeneity in how dietary and exercise adherence were monitored across the included studies. While some trials employed structured dietary records^41^ or 24‐h recalls to assess dietary compliance,^39^ others relied on self‐reported food frequency questionnaires or counselling session attendance,^37^, ^66^ which may introduce varying degrees of reporting bias. Similarly, exercise adherence was inconsistently monitored, with some studies using supervised sessions^21^, ^39^ or wearable devices to ensure protocol fidelity, while others relied solely on participant self‐report.^38^ These methodological differences in adherence assessment may contribute to variability in intervention effectiveness and should be taken into account when comparing outcomes across studies.

### 4.5 Strengths, limitations, and future directions

This review has several strengths, including the inclusion of only studies with RCT design, which represents the highest level of evidence. Additionally, we incorporated meta‐analyses with subgroup analyses to provide a more nuanced understanding of how intervention duration influences the efficacy of LCD + EX interventions in the T2DM population. Our analysis evaluates the overall effect of LCD + EX, irrespective of caloric restriction, emphasizing the specific role of carbohydrate restriction within combined lifestyle intervention. This approach highlights the practical relevance of non‐caloric‐restriction LCDs, which align more closely with real‐world applicability. Compared to traditional caloric‐control diets, non‐caloric‐restriction LCDs demonstrate greater feasibility and adherence among individuals with T2DM, underscoring the practical significance of this study.^67^, ^68^

A key limitation of the present review is the reliance on a relatively small number of studies (*k* < 10) for some outcome analyses. This may be influenced by inconsistent study quality, varying sample sizes, and differing intervention standards, resulting in a relatively very low level of certainty in the GRADE evidence. Nonetheless, we performed sensitivity and heterogeneity analysis in accordance with the established Cochrane guidelines to ensure the robustness of our findings. Our results highlight the need to specify whether total energy, protein, and fat intake are restricted in dietary intervention studies, as well as the need to control for confounding variables in study design to more accurately assess the independent role of carbohydrate intake regulation. Future studies should focus on differences in metabolic outcomes among various types of LCD to explore the application of LCD in preventing and controlling diverse metabolic diseases and provide more robust and conclusive evidence.

## 5 CONCLUSION

In conclusion, the overall results in LCD + EX compared with NRD + EX show no differences in glycemic control and most of the metabolic markers in T2DM. Notably, our findings revealed a modest benefit of LCD + EX for glycemic control in the earlier stage of intervention compared to NRD + EX and selective improvements in the lipid profile. These findings highlight that LCD + EX is a viable approach to non‐pharmacological strategies in T2DM management and emphasize the need for integrating lifestyle changes as a core component of the treatment plan.

## AUTHOR CONTRIBUTIONS

Y.H., S.W., and E.P. conceived the idea for the review. Y.H. and E.P. conducted the literature search, data extraction, statistical analysis, and drafted the manuscript. Z.D. and A.Y. contributed to data verification and provided methodological support. S.W. and E.P. provided critical guidance on review methodology and revised the manuscript for important intellectual content. Y.H., Z.D., A.Y., S.W., and E.P. contributed to writing the manuscript. S.W. and E.P. contributed equally to this work and share corresponding authorship. All authors reviewed and approved the final manuscript.

## FUNDING INFORMATION

This study did not receive any specific grant from funding agencies in the public, commercial, or not‐for‐profit sectors.

## CONFLICT OF INTEREST STATEMENT

The authors declare no conflicts of interest.

## Supporting information


**Data S1.** Supporting Information tables.

## Data Availability

The data that support the findings of this study are available from the corresponding author upon reasonable request.
