# Supplementary material for: Efficacy of a low‐carbohydrate diet combined with exercise on glycemic control and metabolic health in type 2 diabetes mellitus: A systematic review and meta‐analysis
Source: Diabetes Obes Metab. 2025 Dec 19;28(3):1934–46. doi: 10.1111/dom.70379 (PMC12890744; doi:10.1111/dom.70379)
Supplement: Supplementary file 1 — Data S1. Supporting Information tables. [file DOM-28-1934-s001.pdf]

Title: Efficacy of a Low-Carbohydrate Diet Combined with Exercise on Glycemic Control and Metabolic Health in Type 2 Diabetes Mellitus: A Systematic Review and Meta-Analysis
First author: Ye He

Supplementary legend

Supplementary Table 1. Search strategy

Supplementary Table 2. Summary table of the effects of LCD+EX compared with NRD+EX

Supplementary Table 3. Summary table of leave-one-out sensitivity analysis

Supplementary Table 4. Overall certainty of evidence

Title: Efficacy of a Low-Carbohydrate Diet Combined with Exercise on Glycemic Control and Metabolic Health in Type 2 Diabetes Mellitus: A Systematic Review and Meta-Analysis
First author: Ye He

Supplementary Table 1. Search strategy

Database	Search strategy	Results
Cochrane Library	("diet, carbohydrate-restricted" OR "low-carbohydrate" OR ketogenic OR "dietary carbohydrate" OR "low glycemic"):ti,ab,kw AND (exercise OR training OR "physical activity" OR movement OR "exercise therapy" OR "muscle strength" OR "weight lifting" OR "circuit training" OR "resistance training" OR "isometric exercise" OR "aerobic exercise" OR "endurance training" OR "high intensity interval training" OR "moderate intensity interval training" OR "anaerobic training"):ti,ab,kw AND ("randomized control trial")	758
	In Trials (Word variations have been searched); English	
Embase	(("diet, carbohydrate-restricted" or "low-carbohydrate" or ketogenic or "dietary carbohydrate" or "low glycemic") and (exercise or training or "physical activity" or movement or "exercise therapy" or "muscle strength" or "weight lifting" or "circuit training" or "resistance training" or "isometric exercise" or "aerobic exercise" or "endurance training" or "high intensity interval training" or "moderate intensity interval training" or "anaerobic training")).mp. [mp=title, abstract, heading word, drug trade name, original title, device manufacturer, drug manufacturer, device trade name, keyword heading word, floating subheading word, candidate term word]	260
	Filters - Full text, Human, clinical trial or randomized controlled trial or controlled clinical trial and English	
PubMed	(("Diet, Carbohydrate-Restricted"[Mesh]) OR ((((low-carbohydrate) OR (ketogenic)) OR (dietary carbohydrate)) OR (low glycemic))) AND (("Exercise"[Mesh]) OR (((((((((((((training) OR (physical activity)) OR (movement)) OR (exercise therapy)) OR (muscle strength)) OR (weight lifting)) OR (circuit training)) OR (resistance training)) OR (isometric exercise)) OR (aerobic exercise)) OR (endurance training)) OR (high intensity interval training)) OR (moderate intensity interval training)) OR (anaerobic training)))	2768
	Limiters - Full Text, Humans, Randomized Controlled Trial, English	
SPORTDiscus	("diet, carbohydrate-restricted" or "low-carbohydrate" or Ketogenic or "dietary carbohydrate" or "low glycemic") AND (exercise or training or "physical activity" or movement or "exercise therapy" or "muscle strength" or "weight lifting" or "circuit training" or "resistance training" or "isometric exercise" or "aerobic exercise" or "endurance training" or "high intensity interval training" or "moderate intensity interval training" or "anaerobic training") AND (randomized controlled trials or rct or randomized control trials)	26
	Limiters - Full Text, English	

Title: Efficacy of a Low-Carbohydrate Diet Combined with Exercise on Glycemic Control and Metabolic Health in Type 2 Diabetes Mellitus: A Systematic Review and Meta-Analysis
First author: Ye He

Web of Science	(ALL=("diet, carbohydrate-restricted" or "low-carbohydrate" or ketogenic or "dietary carbohydrate" or "low glycemic")) AND ALL=(exercise or training or "physical activity" or movement or "exercise therapy" or "muscle strength" or "weight lifting" or "circuit training" or "resistance training" or "isometric exercise" or "aerobic exercise" or "endurance training" or "high intensity interval training" or "moderate intensity interval training" or "anaerobic training") AND ALL=(randomized controlled trial)<br><br>Limiters – N/A	526

Title: Efficacy of a Low-Carbohydrate Diet Combined with Exercise on Glycemic Control and Metabolic Health in Type 2 Diabetes Mellitus: A Systematic Review and Meta-Analysis
First author: Ye He

Supplementary Table 2. Summary table of the effects of LCD+EX compared with NRD+EX

Outcomes	Number of studies	MD [95% CI]	P	I^2 *(%)*
**HbA1c (%)**	9	-0.18 [-0.43, 0.07]	0.16	59
Duration ≤ 6 months	4	-0.30 [-0.57, -0.03]	**0.03**	41
Duration > 6 months	5	-0.04 [-0.45, 0.37]	0.85	63
**Fasting glucose (mmol/L)**	6	-0.30 [-0.67, 0.07]	0.11	14
Duration ≤ 6 months	3	-0.34 [-0.69, 0.00]	0.05	0
Duration >6 months	3	0.04 [-1.19, 1.28]	0.95	60
**Insulin levels (μIU/mL)**	4	-1.45 [-3.62, 0.73]	0.19	0
**HOMA-IR**	4	-0.17 [-0.46, 0.11]	0.23	0
**Body weight (kg)**	10	-0.46 [-2.07, 1.15]	0.57	45
Duration ≤ 6 months	4	-2.15 [-4.44, 0.14]	0.07	15
Duration > 6 months	6	1.33 [0.51, 2.15]	**0.002**	0
**BMI**	6	-0.54 [-1.51, 0.42]	0.27	43
Duration ≤ 6 months	3	-1.22 [-2.80, 0.36]	0.13	38
Duration > 6 months	3	0.01 [-0.92, 0.94]	0.99	0
**Waist circumference (cm)**	5	-1.01 [-3.50, 1.49]	0.43	0
**Body fat (kg)**	4	0.02 [-2.60, 2.64]	0.99	0
**Fat free mass (kg)**	4	-0.21 [-1.20 to 0.78]	0.68	0
**Systolic blood pressure (mmHg)**	5	-0.36 [-3.89, 3.18]	0.84	0
**Diastolic blood pressure (mmHg)**	5	0.11 [-2.08, 2.30]	0.92	0
**TC (mmol/L)**	5	-0.04 [-0.27, 0.20]	0.75	0
**HDL-C (mmol/L)**	5	0.07 [0.01, 0.14]	0.03	0
**LDL-C (mmol/L)**	5	-0.02 [-0.24, 0.19]	0.84	0
**TG (mmol/L)**	5	-0.23 [-0.43, -0.04]	0.02	0

Title: Efficacy of a Low-Carbohydrate Diet Combined with Exercise on Glycemic Control and Metabolic Health in Type 2 Diabetes Mellitus: A Systematic Review and Meta-Analysis
First author: Ye He

Supplementary Table 3. Summary table of leave-one-out sensitivity analysis

**HbA1c (%)**			
Omit study	MD [95% CI]	I^2 *(%)*	P
Han et al., 2021	-0.12 [-0.38, 0.15]	51	0.38
Kakoschke et al., 2021	-0.28 [-0.48, -0.07]	29	0.009
Kindlovits et al., 2024	-0.16 [-0.44, 0.13]	64	0.27
Rock et al., 2014	-0.11 [-0.37, 0.14]	55	0.38
Struik et al., 2020	-0.21 [-0.50, 0.08]	61	0.16
Tay et al., 2014	-0.14 [-0.40, 0.11]	61	0.27
Tay et al., 2015	-0.19 [-0.46, 0.08]	64	0.16
Tay et al., 2018	-0.21 [-0.46, 0.05]	62	0.12
Wycherley et al., 2016	-0.19 [-0.46, 0.09]	64	0.18
**Fasting glucose (mmol/L)**			
Han et al., 2021	-0.19 [-0.79, 0.40]	30	0.53
Kindlovits et al., 2024	-0.22 [-0.72, 0.28]	29	0.39
Rock et al., 2014	-0.25 [-0.58, 0.09]	1	0.15
Tay et al., 2014	-0.34 [-0.77, 0.09]	22	0.12
Tay et al., 2015	-0.38 [-0.69, -0.06]	0	0.02
Tay et al., 2018	-0.35 [-0.71, 0.00]	9	0.05
**Insulin levels (μIU/mL)**			
Rock et al., 2014	-0.83 [-3.12, 1.46]	0	0.48
Tay et al., 2014	-2.09 [-5.39, 1.21]	21	0.22
Tay et al., 2015	-1.89 [-5.01, 1.23]	26	0.23
Tay et al., 2018	-1.84 [-4.80, 1.13]	26	0.22
**HOMA-IR**			
Kindlovits et al., 2024	-0.10 [-0.39, 0.19]	0	0.50
Tay et al., 2014	-0.25 [-0.69, 0.19]	28	0.26
Tay et al., 2015	-0.24 [-0.66, 0.18]	30	0.26
Tay et al., 2018	-0.23 [-0.64, 0.18]	30	0.26
**Body weight (kg)**			
Brinkworth et al., 2016	-1.21 [-2.58, 0.15]	0	0.08
Han et al., 2021	0.98 [0.22, 1.74]	0	0.01
Kakoschke et al., 2021	-0.61 [-2.51, 1.30]	50	0.53
Kindlovits et al., 2024	-0.52 [-2.23, 1.19]	51	0.55
Rock et al., 2014	-0.47 [-2.19, 1.26]	50	0.59
Struik et al., 2020	-0.33 [-2.17, 1.50]	38	0.72
Tay et al., 2014	-0.47 [-2.15, 1.21]	51	0.58
Tay et al., 2015	-0.58 [-2.35, 1.18]	51	0.52
Tay et al., 2018	-0.53 [-2.28, 1.21]	51	0.55
Wycherley et al., 2016	-0.50 [-2.19, 1.19]	51	0.57
**BMI**			
Han et al., 2021	-0.01 [-0.85, 0.83]	0	0.98

Kindlovits et al., 2024	-0.65 [-1.70, 0.41]	50	0.23
Rock et al., 2014	-0.51 [-1.73, 0.70]	54	0.41
Tay et al., 2014	-0.48 [-1.58, 0.61]	54	0.38
Tay et al., 2015	-0.75 [-1.83, 0.33]	42	0.17
Tay et al., 2018	-0.70 [-1.81, 0.41]	46	0.22
**Body fat (kg)**			
Kindlovits et al., 2024	0.02 [-2.97, 3.01]	0	0.99
Tay et al., 2014	0.38 [-2.39, 3.14]	0	0.79
Tay et al., 2015	-0.34 [-3.58, 2.89]	0	0.84
Tay et al., 2018	-0.12 [-3.29, 3.06]	0	0.94
**Fat-free mass (kg)**			
Kindlovits et al., 2024	-0.17 [-1.18, 0.83]	0	0.73
Tay et al., 2014	-0.23 [-1.24, 0.77]	0	0.65
Tay et al., 2015	-0.22 [-1.64, 1.20]	0	0.76
Tay et al., 2018	-0.22 [-1.53, 1.10]	0	0.75

Title: Efficacy of a Low-Carbohydrate Diet Combined with Exercise on Glycemic Control and Metabolic Health in Type 2 Diabetes Mellitus: A Systematic Review and Meta-Analysis
First author: Ye He

Supplementary Table 4. Overall certainty of evidence

Certainty assessment							№ of patients		Effect		Certainty	Importance
№ of studies	Study design	Risk of bias	Inconsistency	Indirectness	Imprecision	Other considerations	LCD+EX	NRD+EX	Relative (95% CI)	Absolute (95% CI)		
HbA1c (assessed with: %)												
9	randomised trials	serious[a]	serious[b]	not serious	serious[c]	none	271	267	-	MD 0.18 lower (0.43 lower to 0.07 higher)	⨁◯◯◯ Very low[a,b,c]	
Fasting glucose (assessed with: mmol/L)												
6	randomised trials	serious[a]	not serious	not serious	serious[c]	none	193	196	-	MD 0.3 lower (0.67 lower to 0.07 higher)	⨁⨁◯◯ Low[a,c]	
**Insulin levels (assessed with: μIU/mL)**												
4	randomised trials	serious[a]	not serious	not serious	very serious[c,d]	none	119	121	-	MD 1.45 lower (3.62 lower to 0.73 higher)	⨁◯◯◯ Very low[a,c,d]	
HOMA-IR												
4	randomised trials	serious[a]	not serious	not serious	very serious[c,d]	none	67	68	-	MD 0.17 lower (0.46 lower to 0.11 higher)	⨁◯◯◯ Very low[a,c,d]	
Body weight (assessed with: kg)												
10	randomised trials	serious[a]	serious[b]	not serious	serious[c]	none	323	311	-	MD 0.46 lower (2.07 lower to 1.15 higher)	⨁◯◯◯ Very low[a,b,c]	
BMI												
6	randomised trials	serious[a]	serious[b]	not serious	serious[c]	none	204	203	-	MD 0.54 lower (1.51 lower to 0.42 higher)	⨁◯◯◯ Very low[a,b,c]	

Title: Efficacy of a Low-Carbohydrate Diet Combined with Exercise on Glycemic Control and Metabolic Health in Type 2 Diabetes Mellitus: A Systematic Review and Meta-Analysis
First author: Ye He

Certainty assessment							№ of patients		Effect		Certainty	Importance
№ of studies	Study design	Risk of bias	Inconsistency	Indirectness	Imprecision	Other considerations	LCD+EX	NRD+EX	Relative (95% CI)	Absolute (95% CI)		
Waist circumference (assessed with: cm)												
5	randomised trials	serious[a]	not serious	not serious	very serious[c,d]	none	144	142	-	MD 1.01 lower (3.5 lower to 1.49 higher)	⨁◯◯◯ Very low[a,c,d]	
Body fat (assessed with: kg)												
4	randomised trials	serious[a]	not serious	not serious	very serious[c,d]	none	67	68	-	MD 0.02 higher (2.6 lower to 2.64 higher)	⨁◯◯◯ Very low[a,c,d]	
Fat-free mass (assessed with: kg)												
4	randomised trials	serious[a]	not serious	not serious	very serious[c,d]	none	67	68	-	MD 0.21 lower (1.2 lower to 0.78 higher)	⨁◯◯◯ Very low[a,c,d]	
Systolic blood pressure (assessed with: mmHg)												
5	randomised trials	serious[a]	not serious	not serious	very serious[c,d]	none	135	135	-	MD 0.36 lower (3.89 lower to 3.18 higher)	⨁◯◯◯ Very low[a,c,d]	
Diastolic blood pressure (assessed with: mmHg)												
5	randomised trials	serious[a]	not serious	not serious	very serious[c,d]	none	135	135	-	MD 0.11 higher (2.08 lower to 2.3 higher)	⨁◯◯◯ Very low[a,c,d]	
TC (assessed with: mmol/L)												
5	randomised trials	serious[a]	not serious	not serious	very serious[c,d]	none	133	135	-	MD 0.04 lower (0.27 lower to 0.2 higher)	⨁◯◯◯ Very low[a,c,d]	

Title: Efficacy of a Low-Carbohydrate Diet Combined with Exercise on Glycemic Control and Metabolic Health in Type 2 Diabetes Mellitus: A Systematic Review and Meta-Analysis
First author: Ye He

Certainty assessment							№ of patients		Effect		Certainty	Importance
№ of studies	Study design	Risk of bias	Inconsistency	Indirectness	Imprecision	Other considerations	LCD+EX	NRD+EX	Relative (95% CI)	Absolute (95% CI)		
HDL-C (assessed with: mmol/L)												
5	randomised trials	serious[a]	not serious	not serious	serious[d]	none	133	135	-	MD 0.07 higher (0.01 higher to 0.14 higher)	⨁⨁◯◯ Low[a,d]	
LDL-C (assessed with: mmol/L)												
5	randomised trials	serious[a]	not serious	not serious	very serious[c,d]	none	133	135	-	MD 0.02 lower (0.24 lower to 0.19 higher)	⨁◯◯◯ Very low[a,c,d]	
TG (assessed with: mmol/L)												
5	randomised trials	serious[a]	not serious	not serious	serious[d]	none	133	135	-	MD 0.23 lower (0.43 lower to 0.04 lower)	⨁⨁◯◯ Low[a,d]	

CI: confidence interval; MD: mean difference

Explanations

a. Most included studies had some concerns that might affect the results.

b. Substantial heterogeneity was noted.

c. Imprecision due to confidence intervals included potential for important harm or benefit.

d. Small sample size.
